# Increasing the dose of acute rehabilitation: is there a benefit?

**DOI:** 10.1186/1741-7015-11-199

**Published:** 2013-09-10

**Authors:** Ann M Parker, Robert K Lord, Dale M Needham

**Affiliations:** 1 Division of Pulmonary and Critical Care Medicine, Johns Hopkins University School of Medicine, 1830 East Monument St., 5th Floor, Baltimore, MD 21205, USA; 2 Outcomes after Critical Illness and Surgery (OACIS) Group, Johns Hopkins University, 1830 East Monument St., 5th Floor, Baltimore, MD 21205, USA; 3 Johns Hopkins University School of Medicine, 1830 East Monument St., 5th Floor, Baltimore, MD 21205, USA; 4 Department of Physical Medicine and Rehabilitation, Johns Hopkins University School of Medicine, 1830 East Monument St., 5th Floor, Baltimore, MD 21205, USA

**Keywords:** Occupational therapy, Physical therapy modalities, Rehabilitation, Quality of life, Activities of daily living, Mobility limitation, Length of stay

## Abstract

Rehabilitation interventions, including physiotherapy and occupational therapy, can improve patient outcomes; however, the optimal duration and frequency of inpatient rehabilitation interventions is uncertain. In a recent randomized controlled trial published in *BMC Medicine*, 996 patients in two publicly-funded Australian metropolitan rehabilitation facilities were assigned to physiotherapy and occupational therapy delivered Monday through Friday (five days/week control group) versus Monday through Saturday (six days/week intervention group). This increased dose of rehabilitation in the intervention group resulted in greater functional independence and quality of life at discharge, with a trend towards significant improvement at six-month follow-up. Moreover, the length of stay for the intervention group was shorter by two days (95% CI 0 to 4, *P* = 0.10). Hence, in the acute inpatient rehabilitation setting, a larger dose of physiotherapy and occupational therapy, via six versus five days/week treatment, improves patient outcomes and potentially reduces overall length of stay and costs.

Please see related research: http://www.biomedcentral.com/1741-7015/11/198.

## Background

Across a wide variety of settings, physiotherapy (PT) and occupational therapy (OT) can improve patient outcomes, including physical function and quality of life, and reduce length of stay (LOS) and healthcare costs [1-3]. However, the optimal dose of PT and OT has not been established [2], making Peiris and colleagues’ [4] recently reported randomized controlled trial (RCT) comparing six versus five days/week inpatient rehabilitation of great importance.

A prior meta-analysis, including 16 RCTs, demonstrated improved patient outcomes and reduced LOS with extra PT (weighted mean of 19 minutes/day) in acute care and rehabilitation hospitals [2]. While most of the individual RCTs did not show a significant benefit of extra PT, the meta-analysis demonstrated that additional PT resulted in a significant decrease in average LOS of one day in acute care and four days in rehabilitation settings, along with significantly greater functional mobility and quality of life. The RCTs included in the meta-analysis delivered extra PT through a variety of approaches, including both longer duration of sessions and additional weekend or weekday sessions; thus, the relative contribution of these differences in duration and frequency of PT could not be addressed. A better understanding of this issue of rehabilitation dose is important because the delivery of rehabilitation services on weekends varies substantially throughout Australia, Europe and North America, with fewer services frequently provided [2,5-8].

### Description of the RCT

Peiris and colleagues [4] conducted an assessor-blinded RCT including 996 patients in two Australian metropolitan rehabilitation facilities from July 2010 to June 2011. Patients were assigned to one hour each of PT and OT either Monday through Friday (five days/week usual care group) or Monday through Saturday (six days/week intervention group). The extra rehabilitation therapy provided on Saturdays was administered by the patient’s weekday therapist or by another routine care therapist (rather than a research therapist), and in the latter case, the Saturday therapy was based on written handover instructions from the weekday therapist. Non-English speaking patients and those with reduced cognition were included in the study, thus enhancing the generalizability of the results. In addition to evaluating functional independence (measured via the Functional Independence Measure (FIM)), quality of life (via EQ-5D questionnaire) and length of stay (LOS) at discharge, functional independence and quality of life were also assessed at 6- and 12-month follow-up.

While Peiris and colleagues [4] aimed to provide the intervention group with an additional two hours of rehabilitation services on Saturdays, an average of 53 additional minutes of rehabilitation were actually administered during the RCT, with missed therapy sessions resulting from patients feeling unwell, having day leave, or being admitted too late to permit randomization prior to the start of weekend rehabilitation sessions. LOS was non-significantly reduced in the intervention versus usual care group, with a mean LOS of 21 versus 23 days. At discharge, the intervention versus usual care group had greater functional independence and quality of life, with intervention patients being more likely to achieve clinically significant improvements from admission. There were no serious adverse events in either group and no significant difference between groups with respect to any adverse events. At the 6-month follow-up, improvement in patients’ functional independence and quality of life from their status upon admission to the rehabilitation facility still favored the intervention group, but the magnitude of this improvement was smaller, with no significant differences remaining by the 12-month follow-up.

### Interpretation and clinical implications

This RCT has important implications for the delivery of patient care in facilities where rehabilitation services are currently restricted to weekdays. Given the variability in dose and delivery of existing rehabilitation interventions [2,5-8], the study results and their generalizability must be considered in the context of the usual care control group at the study sites, that is, delivering PT and OT for two hours per day, five days per week [9]. This RCT by Peiris and colleagues, when combined with a prior meta-analysis [2], demonstrates that increasing the standard dose of rehabilitation from five to six days/week broadly improves outcomes for a wide range of patients. The potential benefits of seven versus six days/week rehabilitation or increased duration of weekday sessions is an important area of future research.

This study also raises interesting questions regarding how additional PT and OT improve patient outcomes. One compelling mechanism may be a change in patient motivation induced by weekend therapy. To explore this issue, a separate qualitative analysis of a subgroup of patients from this RCT [10] investigated how patients’ experience of rehabilitation differed whether they received five versus six days/week rehabilitation. While patients in both groups perceived their therapists as providing motivation to participate in rehabilitation, those in the intervention group were more likely to view Saturday as a day to work toward improved function rather than a day of rest. Hence, patients in the intervention group may have realized more of their rehabilitation potential due to a greater sense of motivation to achieve rehabilitation goals imparted by the increased presence of PTs and OTs on weekends.

This increase in motivation among patients in the intervention group may have translated to an increase in habitual activity extending beyond the formal additional Saturday sessions to Sunday as well, when no PT and OT interventions were provided for either group. A second, separate analysis of another sub-group of patients revealed that patients in the intervention versus usual care group took significantly more steps and spent more time upright on Sunday as well as Saturday [11]. Thus, the additional rehabilitation may have led to improved functional independence and quality of life through motivating an increase in habitual activity and creating an expectation that patients should be working toward their rehabilitation goals every day. It is uncertain whether a different intervention that focused on this motivational component, such as an educational intervention promoting physical activity seven days per week, would similarly motivate patients and lead to the demonstrated benefit of PT and OT delivered six days/week.

## Conclusion

In conclusion, Peiris and colleagues’ RCT demonstrates that delivering a mean of 53 additional minutes of PT and OT per week, via six versus five days/week of therapy, significantly improves functional independence and quality of life at discharge, and may decrease LOS and provide some prolonged benefit to the six-month follow-up. In addition, we eagerly await the formal economic analysis planned by Peiris and colleagues to determine if the observed patient benefits and reduced LOS are associated with an overall economic benefit, which would make weekend rehabilitation an even more compelling intervention.

## Abbreviations

FIM: Functional Independence Measure; LOS: Length of stay; OT: Occupational therapy; PT: Physiotherapy; RCT: Randomized controlled trial.

## Competing interests

The authors declare that they have no competing interests.

## Authors’ contributions

AMP, RKL and DMN contributed important intellectual content to the manuscript. As first author, AMP was responsible for drafting and final revisions with substantial contributions from RKL and DMN. All authors read and approved the final manuscript.
